# Sandblasting improves the performance of electrodes of miniature electrical impedance tomography via double layer capacitance

**DOI:** 10.1016/j.heliyon.2020.e03652

**Published:** 2020-04-01

**Authors:** Zahra Rezanejad Gatabi, Raheleh Mohammadpour, Javad Rezanejad Gatabi, Mehri Mirhoseini, Mohsen Ahmadi, Pezhman Sasanpour

**Affiliations:** a Department of Biomedical Engineering and Medical Physics, School of Medicine, Shahid Beheshti University of Medical Sciences, Tehran, Iran; b Institute for Nanoscience and Nanotechnology, Sharif University of Technology, Tehran, Iran; c MSEC Department, Texas State University, San Marcos, Texas, USA; d Amol Faculty of Paramedical Sciences, Mazandaran University of Medical Sciences, Sari, Iran; e School of Nanoscience, Institute for Research in Fundamental Sciences (IPM), P. O. Box 19395-5531, Tehran, Iran

**Keywords:** Biomedical engineering, Materials science, Biomedical devices, Surface, Surface coating, Materials property, Physical property, Electrical property, Microstructure, Electrical impedance tomography, Micro EIT, Sandblasting, Double layer capacitance, Gold electrode

## Abstract

Effect of sandblasting of the copper electrode structures before deposition of gold thin film for micro electrical impedance tomography (EIT) system has been studied experimentally. The comparison has been performed on the unmodified copper electrodes and the sandblasted electrodes before deposition of gold layer, using structural analysis while their performance in EIT system has been measured and analyzed. The results of scanning electron microscopy and atomic force microscopy show that the sandblasting of the electrodes results in the deposition of gold film with smaller grain size and uniformly, comparing to the unmodified structure. The measurement of impedance shows that the sandblasting will increase the double layer capacitance of electrode structure which improves the impedance measurement accordingly.

## 1 Introduction

Electrical impedance tomography (EIT) as a non-invasive imaging technique, operates based on the multi-frequency measurement of the electrical impedance between several couples of electrodes [1, 2]. In addition, the EIT technique has the ability to map the electrical properties (conductivity and permittivity) of samples. In this technique, a single frequency, low amplitude excitation current is injected between two electrodes and the voltage at two further electrodes is measured. This four-probe impedance measurement approach introduces a minimized influence of the electrode polarization [3]. EIT as a cost effective and noninvasive technique is safe from ionizing radiation. This technique has been employed in variety of medical applications such as: cancer detection, brain imaging, assessing abdominal bleeding, ventilation imaging, and pulmonary emboli [1, 4, 5].

Using the electrical properties of biological tissues to extract the anatomical and physiological information of the organs is one of the significant applications of the EIT technique. It is a well known fact that due to the difference in the presence of the ions as free charge carriers, some tissues conduct the electricity better than the others [5]. Electrical conductivity and permittivity variation in a tissue are the bases for the EIT image contrast. The impedance measurement of cells was reported by Hober et al. where they measured the electrical conductivity in the center of the cells [6]. Measurement limitations and the lack of micro-manufacturing technologies was a barrier slowing down the impedance-based cell researches prior to 1980s. Later, Giaver and Keese applied electric fields to study the cell spreading and motion in 1984 [7]. The information provided by the electrical properties of biological cells allows for better understanding the complex physiological states of the cells [8]. For example, altered ion channel activities [9] and cytoplasm conductivity [10] might happen in the cells having abnormalities or in bacteria-infected cells.

Characterization of biological cells using the impedance-based techniques requires simple sample preparation and allows for integration into the applications that require unmodified cell retrieval. The impedance-based methods do not require labeling, a significant advantage compared to the traditional methods that implement radioisotope, fluorescent tags, or light absorption [11, 12]. In addition, impedance-based techniques allow for studying the cellular functions including cell morphology, cell movement, cell attachment, cell spreading, cell wounding assay, cell toxicology, and cell signal transduction [13, 14, 15]. Schwan et al. suggested the micro-impedance tomography for the first time [16] which could be employed to study the cellular migration [17].

While miniaturized EIT systems are powerful tools to study the characteristics of tissue and cell structures, their performance is strongly dependent on the electrical characteristics of the electrodes. The impedance of the employed electrodes and the way the electrodes communicate with the cell structure, affect the measurement results. Employing the electrodes with large specific area, increasing the surface roughness, and the mechanisms to better attachment of the cells and tissues to the electrodes, can improve the characteristics of the EIT system. Micro-structured electrodes, in addition to improving the spatial resolution, allow for increasing the surface area and better attachment of the cells to the electrode surface. Surface micro-modification techniques increase the surface area by increasing the surface roughness, resulting in a better cell adhesion [18]. On the other hand, micro-modification of surface affects the electrical parameters of the measurement cell. The relation between the double layer capacitance and the electrode surface structure has been studied before. Surface modification techniques such as coating the electrodes with spongy platinum is reported to significantly increase the effective surface area and the double layer capacitance of the cell [19]. Four-probe electrolytical conductivity measurement cells are currently assumed to have a low sensitivity to disturbances caused by double layer effects [20].

In this study, we have compared the effect of sandblasting of electrodes before deposition of gold thin films in their performance in micro EIT applications. In this regards, two electrode structures for EIT have been fabricated, characterized (structurally) and their performance in impedance measurement have been compared. The results of our study show that the proposed low cost technique could provide electrodes with proper roughness and increase the surface to volume ratio which would be resulted in higher double layer capacitance which improves the EIT characterization of the biological entities accordingly. To the best of our knowledge, for the first time, the present study reports a significant error on the four-probe measurement caused by double layer effect. Sand-blasted electrodes and unmodified electrodes are individually analyzed to quantitatively study and address the effect of double layer capacitance on the precision of four-probe electrolytical conductivity measurement systems of EIT.

## 2 Materials and methods

### 2.1 Fabrication of modified electrodes

Sandblasting as a well-known technique for roughening the surface works through the insertion of rigid ceramic particles on the surface. The injected particles would create deep cleavages on the surface. By pulling out the inserted particles, the surface would be roughened. The primary materials used in the sandblasting process are Aluminum Oxide (Al_2_O_3_), Titanium Dioxide (TiO_2_), Silicon Dioxide (SiO_2_), hydroxyl apatite powders and silicate glass [21].

Using Apparatus Dento-prep (Denmark), copper electrodes were sandblasted by 50μm alumina powder. 50μm alumina powders were obtained from the Merck Co. Ltd. Double distilled water (conductivity of 0.055 μS/cm which corresponds to resistivity ~18 MΩ cm) was used throughout the experiments. A thin gold layer was galvanostatically plated on to the surface of the electrodes using E = +2.4V for 60 s. A stainless steel electrode and a copper electrode were used as the anode and cathode, respectively.

### 2.2 Biological sample (tissue)

The liver tissue of mice was chosen as the samples of our study. Since the tissue structure of the liver is relatively homogenous and isotropic, the effect of position and orientation of electrodes would be minimized [22]. All procedures were performed in accordance with the Shahid Beheshti University of Medical Sciences ethical guidelines. Ethical approval (IR.SBMU.MSP.REC.1396.211) was obtained from the Research Ethics Committee of the School of Medicine, Shahid Beheshti University of Medical Sciences. The mice were sacrificed with cervical vertebrae displacement. All animal care procedures were performed according to the international guidelines on the use of laboratory animals.

### 2.3 Characterization of electrodes

Scanning Electron Microscopy (SEM) images were obtained with VEGA3 TESCAN and Energy Dispersive Spectrometer (EDS) analysis were performed using Sirius SD (Scientific instrument. UK). The atomic force microscopy (AFM) images were performed using Nano Wizard Π (JPK Germany).

### 2.4 EIT system

**Figure 1:**
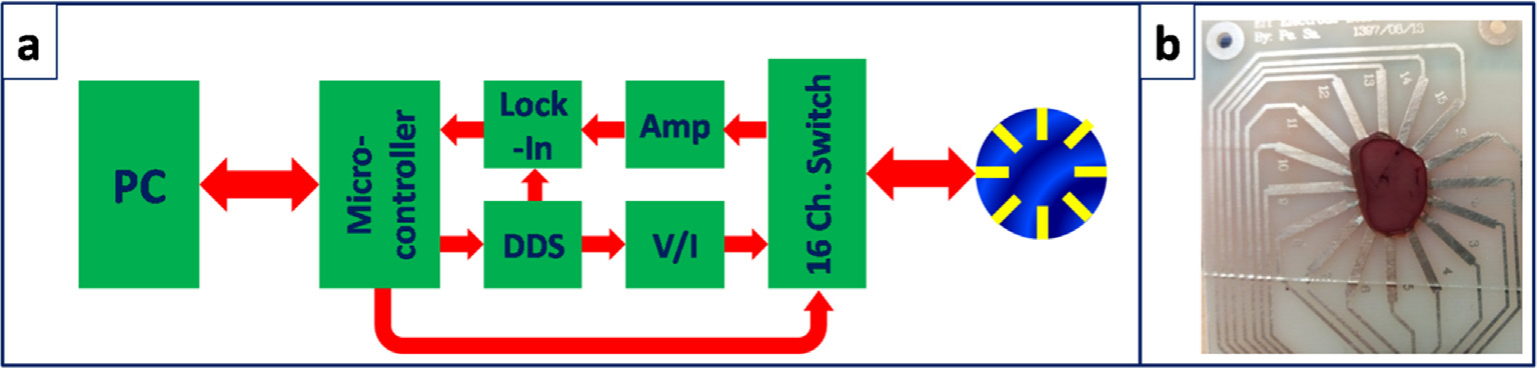
a) Block diagram of the home-made micro EIT system b) The electrode structure board in contact with the liver tissue.

The EIT electrode structure contains of 16-electrodes positioned in a circular pattern. The lengths of the electrodes and spacing between them are identical. All EIT measurements are performed using our own made system with the block diagram of Figure 1-a. The current signal (excitation) is made from a voltage with specific frequency (created by the direct digital synthesizer (DDS) of Analog Devices), through a current-voltage converter. The voltage signal (detection) is measured using high speed FET-Input instrumentation amplifier. The DDS voltage signal and detection signals are fed to the home-made lock in amplifier. The switching between different electrodes for current and voltage are governed through 16 channel analog multiplexer. Control, data acquisition and computer link is performed through a microcontroller. Figure 1-b shows the liver tissue placed on the surface of the electrode structure board, while a microscope cover slip is placed on the tissue to ensure the perfect contact between tissue and electrodes.

### 2.5 Two electrode measurement

In order to find the double layer capacitance of the individual electrodes (two electrode configuration), the measurements are performed using HIOKI3522-50 LCR meter in the frequency range of 0.5Hz–100 kHz.

**Figure 2:**
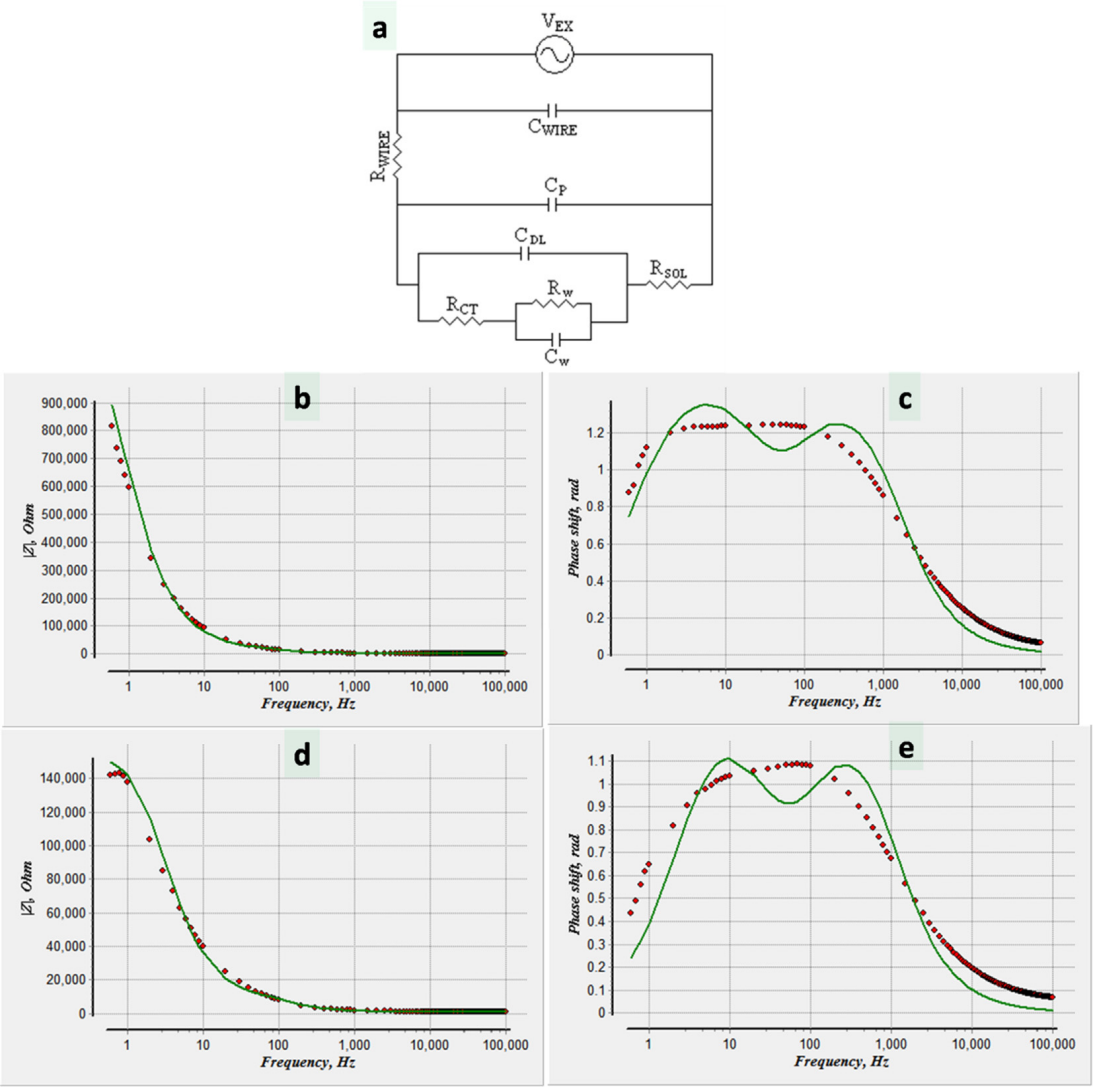
a-Equivalent circuit model of an electrochemical conductivity cell. Absolute value and phase of measured impedance of (b,c) unmodified and (d,e) sandblasted electrode structure.

Figure 2-a illustrates the equivalent circuit of an electrochemical conductivity cell. In addition to the solution resistance (R_SOL_), the circuit contains extra components including: Resistance of the connecting wires (R_WIRE_), capacitance of the connecting wire (C_WIRE_), Faradaic resistance which is also known as charge transfer resistance (R_CT_), double layer capacitance (C_DL_), parallel capacitance of the medium between the electrodes (C_P_), Warburg resistance (R_W_), and Warburg capacitance (C_W_). Figure 2 b, c, d, e shows the measured impedance of unmodified and sandblasted electrodes.

The parallel capacitance of the medium between the electrodes is neglected due to the dimensions of the electrodes and their orientation. R_WIRE_ and C_WIRE_ are compensated prior to the measurements. The modeling and analysis were performed using EIS Spectrum Analyzer software. The results were fitted with the equivalent circuit of electrochemical conductivity and the values of double layer capacitance were extracted and shown in Table 1 accordingly.

**Table 1:** Extracted values of equivalent electric circuit of electrodes (two electrode measurement).

	C_dl_	C_w_	R_sol_	R_ct_	R_w_
Unmodified Electrode	8.73E-08	1.23E-07	1.11E+03	4.16E+04	1.18E+06
Sandblasted Electrode	1.44E-07	3.57E-07	1.07E+03	1.62E+04	1.38E+05

## 3 Results and discussion

**Figure 3:**
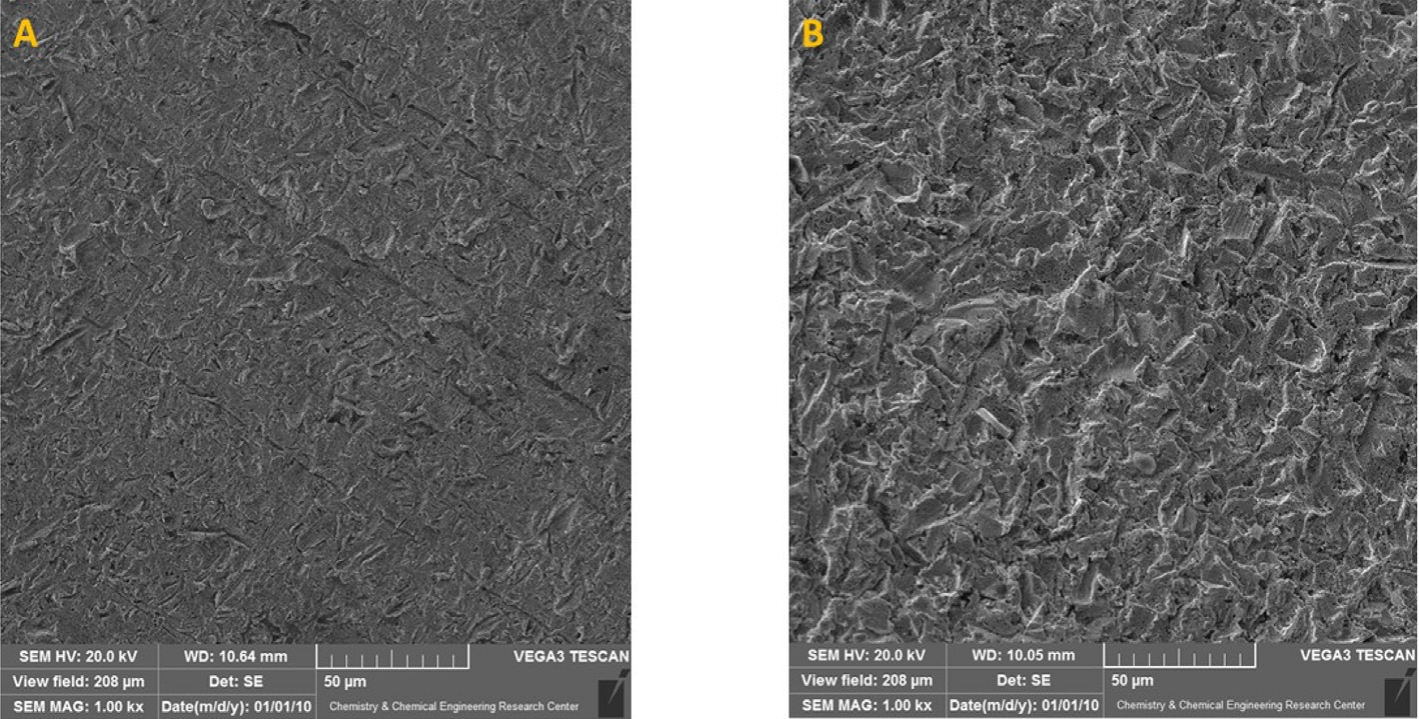
SEM images of A: unmodified electrode and B: sandblasted electrode.

In order to compare the effect of modifying the surface of electrode with sandblast, we have used SEM, EDS and AFM. Figure 3 shows the SEM image of two electrodes. As it can be seen, the sandblasting will make the surface more porous and ragged and the specific surface area will be increased.

**Figure 4:**
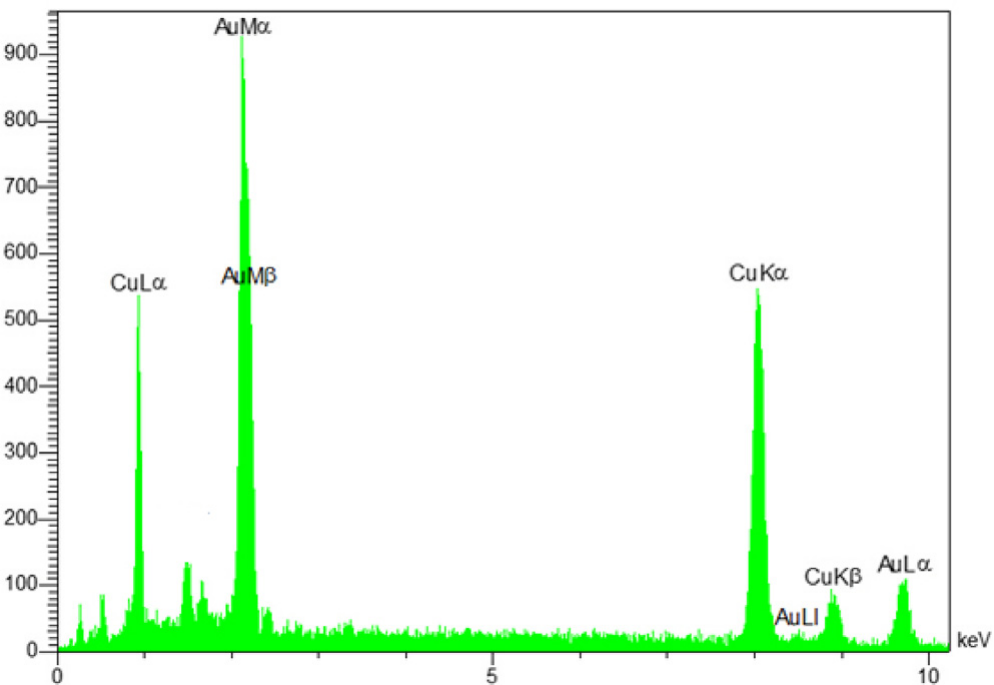
EDS analysis of sandblasted electrode.

Figure 4 shows the EDS analysis of sandblasted electrode which confirms the presence of Cu and Au elements and indicates the deposition of gold layer on the surface of the copper electrodes.

**Figure 5:**
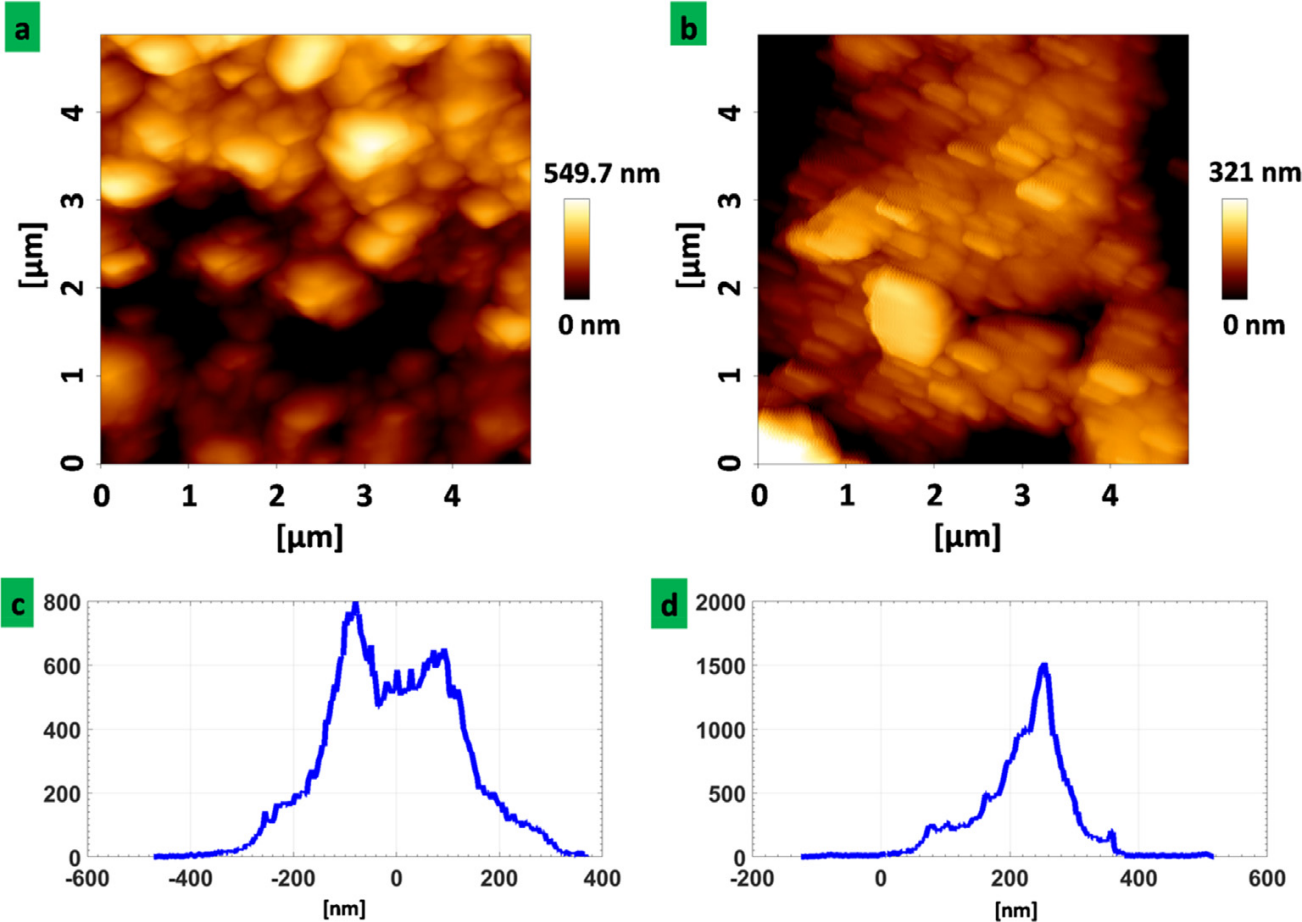
AFM images and height histogram of (a,c) unmodified and (b,d) sandblasted electrode structure.

In order to have a more specific vision on the surface roughness and structure of the surface, Figure 5 shows the AFM images and corresponding height histogram of two electrode structures. For the sandblasted electrode, as the surface is roughened, the deposition will be performed uniformly with small gold nano particles (Figure 5-b) while for the unmodified electrode the deposited surface seem to be covered granular with gold islands (Figure 5-a). The histogram of height for the unmodified electrode (Figure 5-c) shows more broad distribution of heights comparing with the sandblasted electrode structure (Figure 5-d).

**Figure 6:**
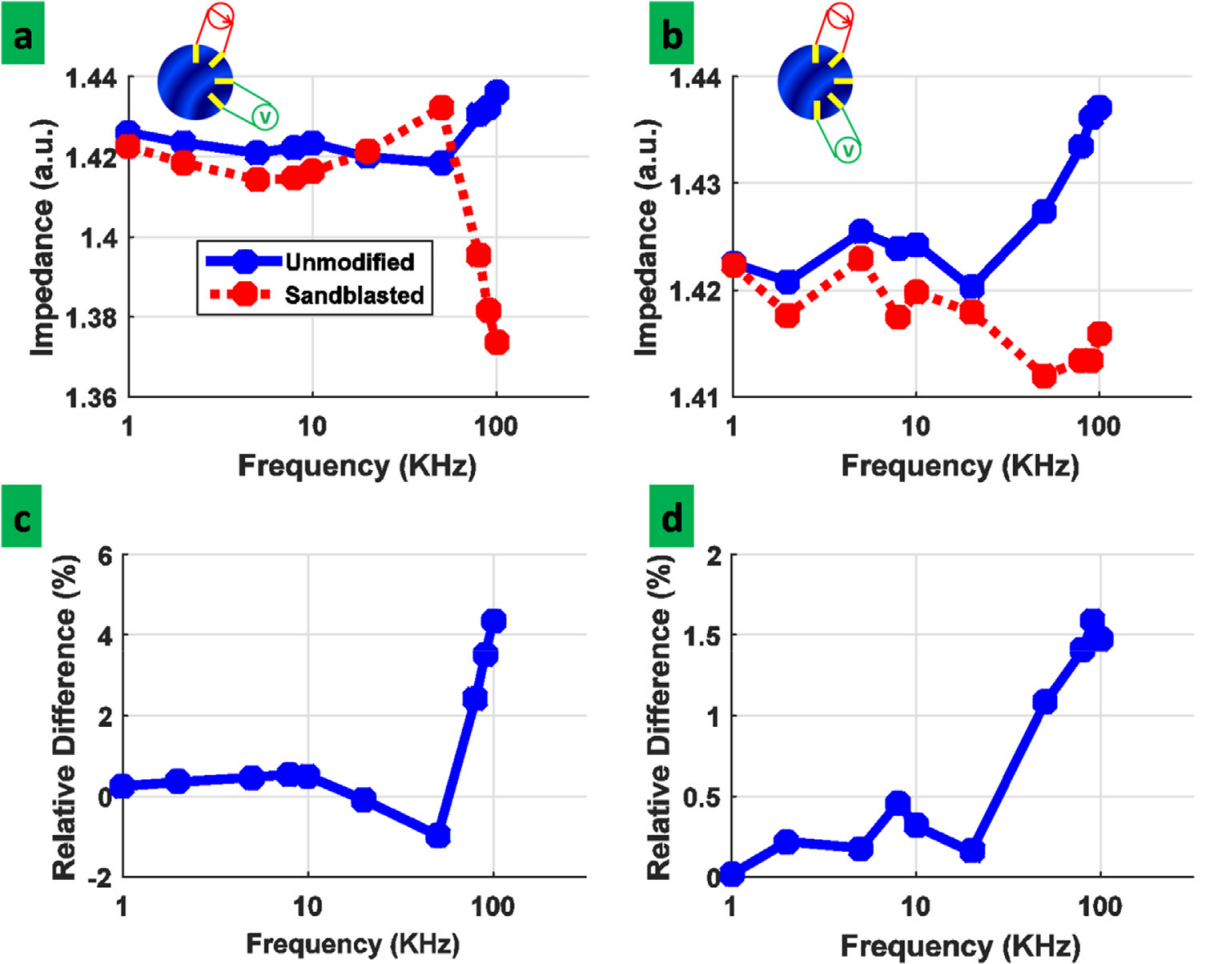
(a,b)- Normalized impedance for unmodified and sandblasted electrode at different configurations (c,d) Relative difference between normalized impedance for unmodified and sandblasted electrodes.

Finally in order to compare the performance of two electrode structures for impedance measurements, the EIT system has been used. The surface of electrodes is covered with the liver tissue (Figure 1-b). Excitation is done through two adjacent electrodes and the recording is performed from two couples of electrodes as shown in subset of Figure 6 (a,b). The results of normalized signal for the two couple of electrodes are shown in Figure 6 (a,b). The signals are recorded in 1, 2, 5, 8, 10, 20, 50, 80, 90 and 100 kHz. Comparing the results for the sandblasted electrode and the unmodified electrodes reveals that there is a difference in the measured impedance, specially in the higher frequency regions. This difference could be associated with the electric double layer capacitance of the electrode structure. As it can be seen in Figure 6(c,d), the relative difference of the measured impedance would reach to the 4.5% at the 100 kHz for adjacent electrode configuration. The sandblasted electrodes based on their porous structure, would present higher capacitance. In order to compare the effect of sandblasting on the double layer capacitance of individual electrodes, two electrode configuration measurements (Table 1) show the value of 8.73×10^−8^ F/cm^2^for the unmodified and the value of 1.44×10^−7^ F/cm^2^ for the sandblasted electrode. The results show the 65% improvement for the individual electrodes. This improvement seems reasonable for the 4.5% improvement in four electrode configuration of measurement. Although in the four electrode configuration the effect of contact impedance would be omitted, the measurements show that the tiny effect of contact impedance would be improved by increasing the double layer capacitance accordingly. The difference behavior of the double layer capacitance for different electrode configurations (Figure 6 a, b) is the direct result of the distance between excitation and recording electrodes. Based on the introduced distance between exciting and recording electrodes, the electric field effect reduces which affects the charge distribution around the recording electrode structure.

## 4 Conclusion

A low cost sandblasting technique for improving the performance of electrode structures for miniaturized EIT system has been proposed, the electrodes have been fabricated and the performances are compared with the untreated ones. The results of our study show that the sandblasting would increase the porosity of the electrode structures which will be resulted in higher values of double layer capacitance. The measurements show that the sandblasted gold coated electrodes structures individually shows 65% improvement in double layer capacitance, which would be resulted in 4.5% of improvement in the EIT system measurements.

## Declarations

### Author contribution statement

Zahra Rezanejad Gatabi: Performed the experiments; Analyzed and interpreted the data; Wrote the paper.

Raheleh Mohammadpour: Conceived and designed the experiments; Analyzed and interpreted the data; Contributed reagents, materials, analysis tools or data; Wrote the paper.

Javad Rezanejad Gatabi & Mohsen Ahmadi: Analyzed and interpreted the data; Wrote the paper.

Mehri Mirhoseini: Contributed reagents, materials, analysis tools or data; Wrote the paper.

Pezhman Sasanpour: Conceived and designed the experiments; Performed the experiments; Analyzed and interpreted the data; Contributed reagents, materials, analysis tools or data; Wrote the paper.

### Funding statement

This research did not receive any specific grant from funding agencies in the public, commercial, or not-for-profit sectors.

### Competing interest statement

The authors declare no conflict of interest.

### Additional information

No additional information is available for this paper.
